# Psychometric evaluation of the Taiwan Chinese version of the EORTC QLQ-PR25 for HRQOL assessment in prostate cancer patients

**DOI:** 10.1186/1477-7525-10-96

**Published:** 2012-08-20

**Authors:** Yu-Jun Chang, Wen-Miin Liang, Hsi-Chin Wu, Hsueh-Chun Lin, Jong-Yi Wang, Tsai-Chung Li, Yi-Chun Yeh, Chih-Hung Chang

**Affiliations:** 1 Graduate Institute of Public Health, China Medical University, Taichung, Taiwan; 2 Epidemiology and Biostatistics Center, Changhua Christian Hospital, Changhua, Taiwan; 3 Graduate Institute of Biostatistics, China Medical University, Taichung, Taiwan; 4 Biostatistics Center, China Medical University, Taichung, Taiwan; 5 Department of Urology, China Medical University Hospital, Taichung, Taiwan; 6 School of Medicine, China Medical University, Taichung, Taiwan; 7 Department of Health Risk Management, China Medical University, Taichung, Taiwan; 8 Department of Health Services Administration, China Medical University, Taichung, Taiwan; 9 Buehler Center on Aging, Health & Society, Northwestern University Feinberg School of Medicine, 750 N. Lake Shore Drive, Suite 601, Chicago, IL, 60611, USA; 10 Division of General Internal Medicine and Geriatrics, Department of Medicine, Buehler Center on Aging, Health & Society, Northwestern University Feinberg School of Medicine, 750 N. Lake Shore Drive, Suite 601, Chicago, IL, 60611, USA

**Keywords:** EORTC QLQ-PR25, Health-related quality of life, Prostate cancer, Rasch analysis, Item response theory

## Abstract

**Objective:**

To evaluate the psychometric properties of the Taiwan Chinese Version of the EORTC QLQ-PR25 health-related quality of life (HRQOL) questionnaire for patients with prostate cancer.

**Methods:**

135 prostate cancer patients were recruited in the urology outpatient clinic of a university teaching hospital. Each patient completed the EORTC QLQ-PR25 at every clinic visit between 2004 and 2008, totaling 633 assessments. Confirmatory factor analysis and Rasch analysis were used to evaluate the domain- and item-level psychometric properties.

**Results:**

The results supported the unidimensionality of each of the four EORTC QLQ-PR25 domains (urinary, bowel, and hormonal-treatment-related symptoms, and sexual functioning). Item calibrations for each domain were found invariant across the three assessment time periods. The item-person maps showed 71.3% of item coverage for the urinary symptoms domain and 13–42.7% for the other three domains.

**Conclusions:**

The Taiwan Chinese Version of the EORTC QLQ-PR25 questionnaire is reliable and can be used to measure HRQOL over time. Adding new items to each domain may improve its clinical content coverage and measurement precision.

## Introduction

Prostate cancer is the most common form of cancer among males in North America and Europe [1], and its incidence rate has increased rapidly in Asia in the past few years [2]. The rate rose from 1.78 per 100,000 persons in 1982 to 24.55 per 100,000 persons in 2008 [3], making prostate cancer a significant public health concern in Taiwan. Although the survival time for patients with prostate cancer has increased due to early detection and improved treatments [4], prostate cancer related symptoms and treatment associated side effects (e.g., urinary, sexual, and bowel dysfunction) have been shown to significantly impact a patient’s health-related quality of life (HRQOL) [4-6]. It is, therefore, important to collect and use reliable and valid patient-reported HRQOL information in order to document their responsiveness to any specific treatments or interventions and to facilitate and guide clinical decisions.

Patient-reported outcomes (PROs), including HRQOL, are becoming increasingly important in clinical research and practice, and therefore much effort has been directed toward the development of more objective methods of assessment [7,8]. For example, the European Organization for Research and Treatment of Cancer (EORTC) Quality of Life Study Group developed the Core Questionnaire (EORTC QLQ-C30) to measure generic aspects of HRQOL for patients with various types of cancer. In order provide more detailed information specific to prostate cancer, a 25-item supplementary module, EORTC QLQ-PR25, was further developed. The EORTC QLQ-PR25 has become a widely used HRQOL questionnaire for prostate cancer patients [9-11]. It includes four domains assessing urinary symptoms, bowel symptoms, treatment-related symptoms, and sexual activity and functioning. The results of international field validation has been published in 2008 [9]. In Taiwan, Chie was authorized by the EORTC to perform Chinese translation (in traditional Chinese characters) and validation the Core Questionnaire (QLQ-C30) and its supplementary modules, and their study results have been in recent years [12-16]. But the psychometric analyses were primarily assessed at the domain level with a small sample size.

In this study, we used the same Taiwan Chinese Version of the EORTC QLQ-PR25 questionnaire published by Chie et al. in 2010 [16] to address issues relevant to traditional psychometric analysis and small sample sizes. We employed both confirmatory factor analysis and Rasch analysis to thoroughly examine and understand its psychometric properties in a larger patient population. Rasch analysis has increasingly become popular in assessing the quality of existing outcome assessment tools and in developing new ones [17,18], as it offers a methodologically rigorous way to evaluate and enhance the measurement properties of the assessment tools [19,20]. Specifically, this study aimed to: 1) examine the stability of item calibrations (i.e., item parameter invariance) within each of the four domains across different time of assessment; 2) evaluate whether the item coverage was adequate to reliably assess the person traits along the latent construct; and 3) determine whether the response category thresholds were in intended sequence (from less to more).

## Methods

### Procedures

A sample of 135 prostate cancer patients treated at the urological department of China Medical University Hospital, a community-based tertiary teaching hospital in Central Taiwan, were recruited from January 7, 2004 to September 15, 2008. This study was approved by the Institutional Review Board of the China Medical University Hospital and all enrolled patients provided written informed consent. Efforts were made to maximize the patient diversity and representativeness to ensure the generalizability of the study results in Taiwan and other Chinese-speaking regions.

Each enrolled patient was asked to do the blood test, biopsies and the Gleason grading system of cells at the baseline assessment. They were also were invited to complete the Taiwan Chinese Version of the EORTC QLQ-C30 (Version 3.0) and the EORTC QLQ-PR25 as well as the amount of prostate-specific antigen (PSA) found in the gland each time during their clinical office visit. During the study period, a total of 633 assessments were collected from these patients at various time points. For the purpose of psychometric evaluation, these assessments were categorized into three groups according to the time of assessment: (1) baseline, pre-treatment (T0, n = 135); (2) one month post first treatment (T1, n = 117); and (3) 3 months or more post treatment (T2, n = 381; some patients completed the EORTC QLQ-PR25 more than once).

### Measure

The EORTC QLQ-PR25 is a 25-item prostate cancer specific HRQOL questionnaire developed by the European Cancer Research and Treatment Organization (EORTC). It has four domains, each with various number of items: Urinary Symptoms (9 items; labeled US31-US39), Bowel Symptoms (4 items; BS40-BS43), Hormonal-treatment-related Symptoms (6 items; TS44-TS49) and Sexual Activity and Functioning (6 items; SX50-SX55) [9]. For this study, 5 items lacking adequate number of responses were excluded from the analysis. One Urinary Symptoms item that did not apply to all patients was excluded (US38, “*Has wearing an incontinence aid been a problem for you? Answer this question only if you wear an incontinence aid.*”). Four follow-up questions (SX52-SX55) to the Sexual Functioning item (SX51, “To what extent were you sexually active?”) were also excluded because about two-thirds of the patients responded not being sexually active. Each of the 20 retained items was rated on a 4-point Liker-type scale (*1 = “Very much”, 2 = “Quite a bit”, 3 = “A little”, 4 = “Not at all”).* To facilitate ease of interpretation and understanding of the analysis results, each item was scored in the same direction, from 1 to 4, with higher scores indicating better HRQOL (i.e., less symptomatic or better functioning).

### Data analysis

Descriptive statistics (e.g., mean, standard deviation, frequency, etc.) were calculated using SPSS version 15.0 (SPSS, Inc., Chicago, Illinois) for each item. Cronbach’s coefficient alpha was also calculated for each domain, with a value of 0.7 or higher indicating good internal consistency [21].

A series of confirmatory factor analysis (CFA) using LISREL version 8.72 (Scientific Software International, Inc.) were performed to examine the domain unidimensionality. Unidimensionality, the measurement of one underlying construct and an important pre-requisite for Rasch analysis, was determined by the magnitude of item factor loadings, with a value > 0.3 as an indicative of acceptable item-domain membership. Model fits were considered acceptable, if the goodness-of-fit index (GFI), Bentler-Bonett normed fit index (NFI), non-normed fit index (NNFI) and comparative fit index (CFI) values exceed 0.9, the root mean square error of approximation (RMSEA) values were below 0.05, and the standard root mean-square residual (SRMR) values were below 0.08 [22,23].

Rasch Rating Scale model [24] suitable to calibrate items with ordered response categories (e.g., “Not at all” to “Very much”) as in the EORTC QLQ-PR25 items was used. Item fit, item stability, coverage of item content and person measure (item-person map), and item targeting were evaluated. All Rasch analyses were performed using the Winsteps software, version 3.47 [25].

#### Item difficulty and person measure

In Rasch measurement models, items and persons are jointly place on the same interval-level “logit” metric. A symptom item with a higher item difficulty value as modeled is said to be more ‘difficult’ to not occur (i.e., easier to occur). A functioning item with a higher item difficulty value, however, would be more difficult for a person to respond with a more frequent or severe response (e.g., “very much”) to the function or activity described in that item. Similarly, a person with a higher person measure is ‘more likely’ to choose a less-frequent (e.g., “not at all”) response for a symptom but ‘more likely’ to choose a more-frequent response (e.g., “very much”) for performing that function or activity.

#### Item fit

Item fit was evaluated using the infit mean-square (denoted as Infit) statistic derived from the Rasch analysis. This goodness-of-fit statistic, defined as the ratio of the observed to the predicted variance for an item, indicates how well the item fits the rest of the items in the same domain/scale. An item with an Infit statistic > 1.4 or < 0.7 was considered to be lack of fit according to the Rasch model [8,23,24].

#### Stability of item calibrations

Differential item functioning (DIF), referred to as an item lacking item parameter or measurement equivalence across different groups or settings, was identified statistically by conducting the *t*-tests on the mean item calibrations between the 3 assessment time groups (T0 vs. T1 vs. T2). An item was said to exhibit DIF, if the *t*-test statistic exceeds *p* < 0.016 (= 0.05 / 3) after the Bonferroni correction for multiple testing was applied [26,27]. Once item stability of scaling, i.e., items across group exhibiting no DIF, was achieved, the data from all the three groups were pooled together for subsequent Rasch analyses to assess the item-person coverage and other psychometric properties.

#### Item-person map

The item-person map, where the item difficulties and person measures were plotted together along the same logit scale, is useful for visual inspection to identify areas or gaps along the latent trait continuum that may be lacking items. A logit is the natural log-odds of person being successful at a task (e.g., answering an item correctly) versus those being unsuccessful (e.g., answering an item incorrectly). In this study, it is a unit of measurement to report relative differences between person measure estimates and item difficulties. An item with positive logit value indicates that the item requires a greater level of person measure than the average (i.e., the item is relatively harder). A person with positive logit value indicates that his/her person measure is greater than the mean required level of measure for the items (i.e., the latent trait a person possesses is greater than the overall trait required for the tasks) [28].

Since each item was rated on a 4-point response category (e.g., 1 = “Very much” to 4 = “Not at all” for the frequency of symptoms or functioning), three thresholds (each between the two adjacent response categories as modeled) were obtained. The ordering of the three threshold parameters for each item was examined to assess model fit. It was considered to be in a non-logical sequence when the value of threshold was not shown from less (the first threshold) to more (the third threshold) for each item [29,30].

#### Item coverage of the construct and ceiling or floor effect

The item coverage of the latent construct (e.g., Urinary Symptoms) being measured was defined as the percentage of persons whose person measures fall between the highest and lowest thresholds of all items in that domain. The ceiling effect was defined as the percentage of people whose person measures were greater than the highest (i.e., the third) threshold. Similarly, the floor effect is the percentage of persons whose person measures were less than the lowest (i.e., the first) threshold [29,31]. These three indices were used to determine how well the items in the same scale are targeted to the persons being measured [32,33].

## Results

### Characteristics of the study participants

The demographic and clinical characteristics of the study participants by group (i.e., time of assessment) and as a whole are summarized in Table 1. The mean age of the prostate cancer patients at pre-treatment (T0) was about 70 years. Most patients (67.2%) were in Stage II. Surgery was the most common treatment type followed by radiation therapy. The mean prostate-specific antigen (PSA) values were 46.3, 10.9, and 2.8 for T0, T1, T2, respectively. At T0, the mean Gleason scores were 6.59.

**Table 1:** Demographic and clinical characteristics of the study participants by time of assessment

		**Therapy period**							
		**Before therapy (T0)**		**1 month after therapy (T1)**		**≧3 months after therapy (T2)**		**Total**	
		**(n = 135)**^**a**^		**(n = 117)**^**b**^		**(n = 381)**^**c**^		**(n = 633)**^**d**^	
		**N**	**%**	**N**	**%**	**N**	**%**	**N**	**%**
Age	Mean ± SD	70.0 ± 7.6		70.4 ± 7.6		70.9 ± 7.1		70.6 ± 7.3	
	Median (Range)	70.1 (48.8-90.8)		70.7 (48.8-92)		70.2 (49-93)		70.2 (48.8-93)	
Treatment	Surgery	57	42.2	48	41.0	162	42.6	267	42.2
	Irradiation	38	28.1	35	29.9	156	41.1	229	36.2
	Hormone therapy	31	23.0	31	26.5	60	15.8	122	19.3
	Chemical therapy	9	6.7	3	2.6	2	0.5	14	2.2
PSA	Mean ± SD	46.3 ± 126.9		10.9 ± 33.5		2.8 ± 16.6		12.8 ± 61.7	
PSA group	<=2.5	32	27.8	66	64.7	304	83.1	402	69.0
	2.501-4	6	5.2	4	3.9	21	5.7	31	5.3
	4.001-6	9	7.8	8	7.8	14	3.8	31	5.3
	6.001-10	24	20.9	4	3.9	14	3.8	42	7.2
	>10	44	38.3	20	19.6	13	3.6	77	13.2
Gleason score	2-4	17	14.2	13	12.1	53	14.9	83	14.3
	5-7	70	58.3	58	54.2	203	57.2	331	56.9
	> = 8	33	27.5	36	33.6	99	27.9	168	28.9
Stage	I	1	0.8	1	0.9	4	1.1	6	1.0
	II	84	67.2	75	67.0	276	75.8	435	72.4
	III	20	16.0	21	18.8	57	15.7	98	16.3
	IV	20	16.0	15	13.4	27	7.4	62	10.3

PSA, prostate-specific antigen.

^a^ One hundred and thirty-five assessments were obtained from 135 independent subjects.

^b^ One hundred and eleven assessments were obtained from 117 independent subjects.

^c^ Some subjects (n = 102 subjects) responded to more than one assessment.

^d^ A total of 633 assessments were included in the final model analysis.

Age, main type of treatment and PSA values were assessed at the same time as HRQOL questionnaire administration.

### Descriptive statistics and scale reliability

Descriptive statistics calculated from their raw responses and item statistics derived from the Rasch analysis are shown in Table 2. Items in their respective domain are listed in descending order of their mean raw scores. As can be seen, very high percentages of patients selected the less-frequent ‘Not at all” response (81.8% - 93.5% for the Bowel symptom (BS) items; 52.6% - 96.0% for the Treatment-related symptom (TS), indicating that the majority of patients did not experience these symptoms. In contrast, the Sexual functioning (SX) domain had relatively high percentages (58.5% - 70.8%) of patients choosing the “Not at all” response category, indicating that most patients did not have sexual activity. Only the Cronbach’s coefficients alpha of Urinary symptoms (US) and Sexual functioning (SX) domains were above the acceptable level of 0.7.

**Table 2:** Results of descriptive and psychometric analyses of the EORTC QLQ-PR25 by group

		**Raw responses (n=633)**				**Rasch analysis**								
	**EORTC QLQ-PR25 Items ranked by difficulty**	**Mean score**	**“Very Much”**	**“Not at all”**		**T0**	**T1**	**T2**	**Total**			**DIF (P-value) **		
					**α**	**(n=135)**	**(n=117)**	**(n=381)**	**(n=633)**	**Infit**	**Targeting**	**among Groups: T0, T1, T2**		
		**(SD)**	**%**	**%**		**δ**	**δ**	**δ**	**δ**		**(SD)**	**T0 vs. T1**	**T0 vs. T2**	**T1 vs. T2**
**Urinary symptoms (US)**^**a**^					0.77						2.26			
US37	Painful voiding (least frequent)	3.84(0.39)	0.0	84.5		−1.67	−2.05	−2.10	−2.00	1.23	(1.57)	0.257	0.130	0.865
US39	Limitation of activities because of US	3.67(0.62)	1.8	73.5		−1.30	−0.85	−0.98	−0.72	1.41		0.109	0.185	0.556
US35	Need to remain close to toilet	3.54(0.72)	2.6	64.3		−0.57	−0.21	−0.30	−0.37	1.08		0.147	0.208	0.633
US36	Urinary incontinence	3.53(0.63)	1.0	59.7		−0.91	−0.06	−0.23	−0.35	1.11		0.001	0.002	0.389
US34	Sleep deprivation because of US	3.25(0.82)	4.8	44.2		0.85	0.63	0.54	0.58	1.03		0.291	0.080	0.644
US31	Frequent urination in daytime	3.18(0.79)	3.4	38.7		0.87	0.80	0.83	0.78	0.86		0.753	0.815	0.884
US33	Urinary urgency	3.15(0.81)	6.1	35.7		0.97	0.78	0.94	0.87	0.93		0.347	0.845	0.347
US32	Nocturia (most frequent)	3.01(0.81)	5.3	28.1		1.49	1.09	1.27	1.22	0.79		0.044	0.172	0.278
**Bowel symptoms (BS)**^**a**^					0.41						3.58			
BS42	Fecal blood (least frequent)	3.93(0.31)	0.3	93.5		−0.74	−0.56	−0.77	−0.71	1.27	(1.03)	0.713	0.954	0.622
BS41	Fecal incontinence	3.92(0.29)	0.0	92.6		−0.63	−1.07	−0.47	−0.63	0.98		0.384	0.689	0.172
BS40	Limitation of activities because of BS	3.82(0.45)	0.6	84.8		0.36	0.95	0.51	0.59	0.98		0.101	0.646	0.131
BS43	Bloated feeling (most frequent)	3.80(0.44)	0.5	81.8		0.94	0.50	0.74	0.74	0.95		0.206	0.490	0.421
**Treatment-related symptoms (TS)**^**a**^					0.41						3.32			
TS45	Breast tenderness (least frequent)	3.96(0.20)	0.0	96.0		−1.95	−2.69	−1.42	−1.69	0.96	(1.22)	0.407	0.356	0.098
TS44	Hot flushes	3.89(0.34)	0.0	89.9		−0.46	−0.41	−0.68	−0.57	0.97		0.896	0.514	0.407
TS46	Swelling in legs or ankles	3.84(0.48)	1.0	87.1		0.08	0.25	−0.28	−0.09	1.27		0.599	0.202	0.048
TS48	Bothered due to weight gain	3.81(0.47)	0.6	83.9		−0.07	0.09	0.13	0.09	0.99		0.639	0.483	0.883
TS47	Bothered due to weight loss	3.78(0.51)	0.8	81.8		0.71	0.47	0.04	0.28	1.05		0.394	0.006	0.081
TS49	Felt less masculine (most frequent)	3.31(0.86)	4.9	52.6		1.73	1.76	2.11	1.98	0.97		0.858	0.033	0.045
**Sexual functioning (SX)**^**b**^					0.80						−6.41			
SX50	Sexual interest (more likely)	1.53(0.72)	1.7	58.5		−1.11	−1.67	−1.87	−1.67	0.88	(3.97)	0.314	0.072	0.663
SX51	Sexual activity (less likely)	1.34(0.59)	1.1	70.8		1.10	1.67	1.89	1.67	0.92		0.316	0.068	0.650

Group T0: before therapy; Group T1: one month after the start of therapy; Group T2: therapy for 3 months or more; Group Total: Group T0 + T1 + T2.

^a^ Raw responses: 1: “Very much”; 2: “Quite a bit”; 3: “A little”; 4: “Not at all”, higher score means better HRQOL/less frequent symptom.

^b^ Raw responses: 4: “Very much”; 3: “Quite a bit”; 2: “A little”; 1: “Not at all”, higher score means better HRQOL/more interest/activity.

δ: Item difficulty, higher difficulty means worse HRQOL/more frequent symptom/problem; Targeting: the average person ability level.

α: Cronbach’s α > 0.7, acceptable reliability; Infit statistics > 1.4: item misfit; DIF: differential item functioning.

### Unidimensionality assessement

The results of CFA, after adding some covariances between error terms (3 covariance modifications for the US domain, one covariance modification for the BS domain, and one covariance modification for the TS domain), supported the assumption of unidimensionality as the US, BS and TS domains had GFI, NFI, NNFI and CFI values over 0.9, RMSEA and SRMR values were less than 0.5. The unidimensionality of the 2-item SX domain was evidenced by its Cronbach’s coefficient alpha of 0.8.

### DIF analysis for testing stability of item across groups

As can be seen in Table 2, the sequence of the mean Rash-calibrated item difficulties was consistent across the 3 groups, suggesting that the hierarchical structure of the item difficulty was invariant. Moreover, the ordering of item difficulties (from less to more) in their respective domain was also consistent between the mean raw scores and the Rasch-derived mean item difficulties.

Among all the possible paired comparisons of item calibrations for the three time assessment time points, only three items were found to exhibit DIF. The US item “Urinary incontinence” displayed significant DIF twice: between groups T0 and T1 (*p* = 0.001) and between groups T0 and T2 (*p* = 0.002). The TS item “Bothered due to weight loss” also displayed significant DIF between groups T0 and T2 (*p* = 0.006). As the DIF analysis results supported the stability of item calibrations, it seemed safe to combine the item response data from the three groups (Group T0 + T1 + T2) to obtain the final calibration. The item hierarchy of the combined “Total” group was quite similar to those of the three separate groups (Table 2). Only one US item has a fit statistic greater than pre-specified cut-off value of 1.4, supporting its overall model fit.

### Item-person maps

**Figure 1:**
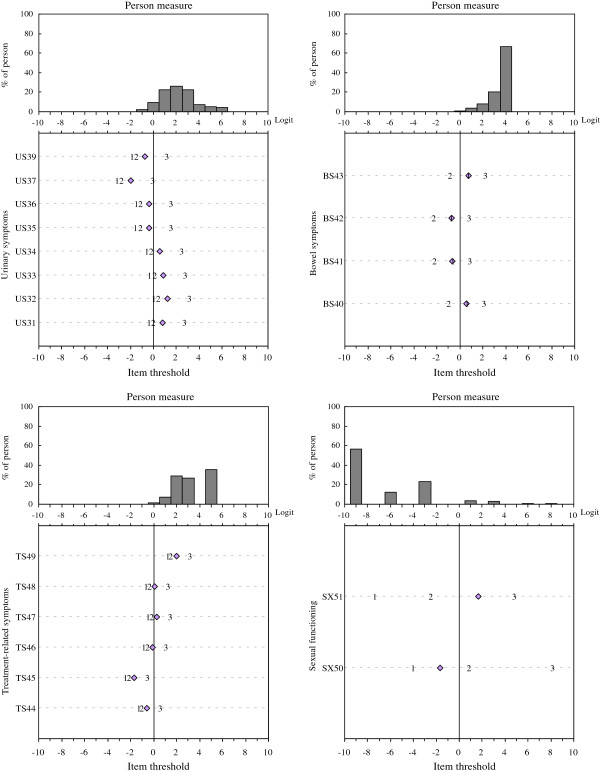
**Person measure-item threshold map for each EORTC QLQ-PR25 domain.** Logit values of the three thresholds and mean item difficulty are represented by the numerical values 1, 2, and 3 and a diamond marker, respectively.

Figure 1 shows the four item-person maps, one for each domain, depicting the person measures (upper panel) and sets of threshold parameters (represented by the numbers 1, 2, 3 for each item in the lower panel) jointly positioned on the same ‘logit’ continuum ranging from −10 to 10 using the pooled data. The average item difficulty is standardized with a mean of 0 and a standard deviation of 1. The number “1” in the lower panel indicates the location at which a patient had a 50% chance to choose either the “Quite a bit” or “Very much” response category. Similarly, the number “2” indicates the level at which a patient had a 50% chance to choose either the “A little” or “Quite a bit” response category. Thus, a patient with an estimated person measure greater than 4 (in logit unit) in the US latent continuum would be more likely to select the “Not at all” response category for all the Urinary Symptom items. Inspection of the ordering of the three thresholds revealed that, a consistent 1-2-3 (less-to-more) order in these domains was observed, except that the Bowel Symptoms domain had an unexpected order sequence (2-1-3 instead of 1-2-3).

Item-person maps are used to see how well the domain-specific items match up with the persons being measured. As can be seen in Figure 1, person measures were distributed more on the right side when compared to the spread of all the item thresholds for the US, BS, and TS domains. This suggested that items in these domains had a greater tendency of a ceiling effect. However, different pattern was observed in the SX domain. A high proportion of the person measures were on the left side, suggesting a floor effect.

### Coverage of the construct for person traits

The range of item thresholds and person measures across the four domains are shown in Table 3. As can be seen, the range of the item thresholds for the EORTC QLQ-PR25 did not fully overlap with the range of the person measures (coverage rate ranging from 13.0% to 73.1%). For the BS and TS domains, the coverage rate was just 13% and 38.5%, respectively. Many patients had person measures greater than the third threshold (87.0% and 61.5% for BS and TS domains, respectively), suggesting a significant ceiling effect (reporting no symptoms) in these two domains. For the SX domain, the coverage rate was 42.7%. 56.9% of the patients whose person measures were less than the lowest threshold, suggesting a significant floor effect (reporting no interest/activity) in the SX domain. For the US domain, the coverage rate was 73.1%. No floor effect was found and the ceiling effect was relatively low at 26.9%.

**Table 3:** The Range of Item Thresholds and Person Measures of the EORTC QLQ-PR25 by Domain

	**US**	**BS**	**TS**	**SX**
Item threshold	-3.15−3.12	-2.26−2.27	-2.41−3.16	-7.33−8.16
Ability	-2.83−5.52	-0.21−4.23	-0.10−4.72	-9.07−8.24
Cover %^a^	73.1	13.0	38.5	42.7
Floor effect	0.0	0.0	0.0	56.9
Ceiling effect	26.9	87.0	61.5	0.5

US, Urinary symptoms; BS, Bowel symptoms; TS, Treatment-related symptoms; SX, Sexual functioning.

^a^ Item threshold range covered person measure percentage.

## Discussion

The results of our analyses of the Taiwan Chinese Version of the EORTC QLQ-PR25 showed that each of the four domains satisfied the unidimensionality assumption and items in their respective domain had a good fit to the Rasch model. Overall, the item hierarchy was found to be consistent and item stability (item parameter invariance) was observed in all four domains across the three time periods. The items in the US domain spread satisfactorily along the latent trait continuum (coverage rate, 71.3%). The significant ceiling effect in both the BS and TS domains, as well as the noticeable floor effect in the SX domain together suggested the inadequate item coverage at the end in these three domains. The ordering of the thresholds for all the domains, except for the BS domain, was in sequence from less to more as intended.

Our findings of low alpha coefficients in the BS and TS domains of the EORTC QLQ-PR25 are similar to those have been previously reported. The EORTC official version [9], Spanish version [10] and the Taiwan Chinese version [16] all reported low reliability (< 0.6) and high ceiling effects in the BS and TS domains. van Andel et al. indicated Cronbach’s alpha coefficient reflects the ratio of variances of the individual scale items to the variance of the total scale. A restricted range of responses will have a greater impact on the total scale score than on the individual items, resulting in a lower reliability estimate [9].

The initial development of the EORTC QLQ-PR25 items nearly 20 years ago was based on the selection of important items by both prostate cancer patients and clinicians. However, recent improvements in treatment (e.g., three-dimensional conformal radiotherapy, intensity-modulated radiotherapy [34]) and symptom control for prostate cancer may have contributed to the low symptom frequency in the items assessed in the BS and TS domains. For example, over 90% patients reported not having “Fecal blood”, “Fecal incontinence” or “Breast tenderness”. In this study, the large ceiling effect (87% and 61.5% in the BS and TS domains, respectively) is consistent with the findings previously reported [9,10]. These items may not seem as important or relevant as previously selected and should be considered for revision or even remove. More clinically relevant items based on clinicians’ recommendations to measure the contemporary patient's symptoms and concerns should be developed and added. For example, items like “Difficulties with bowel function” may be added to the BS domain and “Bone dysfunction” (osteoporosis, etc.) may be added to the TS domain. Adding and validating new clinically relevant and psychometrically sound items in future studies can potentially eliminate the ceiling or floor effect and item content gaps, and may improve the performance of the items within the same domain.

Sexual dysfunction can occur and impact patients regardless of the treatment modalities they receive [35,36], and the time to recover from it is usually much longer than that from other side effects. In general, about 38% to 48% of patients had not recovered from sexual dysfunction one year after receiving treatment [6]. Although there are six SX items in the EORTC QLQ-PR25, four of them (SX52-SX55) are conditional and only applicable to those being sexually active, which may lead to less precise measurement in this domain [30]. Adding more commonly experienced sexual functioning items, such as the impact of “Loss of libido”, may improve it measurement precision and clinical relevance. Using items from other questionnaires, such as the 15-item version of the International Index of Erectile Dysfunction (IIEF-15) [37] and the Male Sexual Health Questionnaire (MSHQ) [38], may help physicians to better measure and monitor changes of the sexual functioning aspect of their patients’ HRQOL.

The first two thresholds were very close to each other but far away from the third threshold, as shown in the item-person maps of the US and TS domains, seemed to suggest that a binary 2-category response category may be practical to improve readability and measurement precision [39]. Furthermore, the issue related to the out-of-sequence thresholds in the BS domain and the noticeable item coverage gaps in the SX domain suggest that further improvement is still needed. Hsueh et al. reported that the middle categories were never the most likely responses of any patient and were thus redundant, when polytomous items of index exhibited disordering of the step difficulty. The psychometric properties of the dichotomous items were equivalent to those of the polytomous items. A scale with only dichotomous items is much more convenient and efficient to administer [40]. Maio and Perrone pointed out that HRQOL assessment in the elderly is complicated by several unresolved methodological problems (higher frequency of illiteracy, worse compliance with the questionnaires, concomitant diseases, use of instruments not validated in the aged population) [41]. A binary response category (“No” for “Not at all” vs. “Yes” for combined “A little”, “Quite a bit” and “Very much”) may be practically feasible to improve readability and measurement precision for the Taiwan Chinese Version of the EORTC QLQ-PR25 and easier to respond for prostate cancer patients, who are typically older (70% of all prostate cancers are diagnosed in men over the age of 70 in Taiwan) [3] and less educated (half of the patients had an education level of less than 9 years in this study) [42].

Besides the many statistics and ways to allow for thorough psychometric evaluation from the Rasch analysis in this study, one additional strength is that our data were from a large group of prostate cancer patients with varying levels of severity, receiving different treatment modalities and assessed at various times. As shown in Table 2, the assessments were grouped into three groups based on the assessment time period. The data were first analyzed separately by time period to validate the EORTC QLQ-PR25. The stability of item calibrations within each domain was then compared across different time periods. The data of these three periods were then combined and validate again. The combined data potentially maximize the patient diversity and representativeness to ensure the generalizability of the study results in Taiwan. Our results showed the item stabilities held across the three different time periods, satisfying an important measurement property for making meaningful HRQOL score comparison for prostate cancer patients in a longitudinal study [43].

Some limitations of this study should also be noted. First, this study was limited in scope only to the stability assessment across different time periods; therefore larger-scale studies with adequate sample sizes are still needed to examine the stability of item calibrations across different age groups, cancer stages, and treatment groups. Secondly, only outpatients in Central Taiwan were sampled, which might limit its generalizability to all Chinese-speaking prostate cancer patients in Taiwan or other regions. Patients coming to outpatient clinics normally are expected to have milder symptoms than those in the inpatient settings, and may produce a higher ceiling effect in our study. Thirdly, this study did not include stratified analysis of different types of treatment and disease stages. However, since patients with prostate cancer often receive multiple treatment modalities and exhibit long disease duration, our study cohort appears to be a fair representative and therefore the results from this pooled sample can be of practical value for clinical implications.

## Conclusions

The domain-specific items in the EORTC QLQ-PR25 were found to be unidimensional in measuring their intended domain. The differential item functioning analysis results indicated that item calibrations were stable across different time periods for each of the four EORTC QLQ-PR25 domains. Adding more clinical relevant and content appropriate items to each domain to fill the item coverage gaps as well as to eliminate the ceiling and floor effects would be desirable, so that the content relevance and scale performance can be improved. Our systematic and thorough analysis steps to better understand the psychometric properties of the Taiwan Chinese Version of the EORTC QLQ-PR25 may be used to identify what can and should be done for questionnaire development. By using a clinically relevant, psychometrically sound, and culturally appropriate assessment tool, clinicians can adequately and accurately assess the HRQOL and the impacts of treatment on their patients to plan for better treatment strategies.

## Abbreviations

EORTC: The European Organization for Research and Treatment of Cancer; EORTC QLQ-PR25: EORTC quality of life questionnaire prostate-specific 25-item; HRQOL: Health-related quality of life; PCa: Prostate cancer; PROs: Patient-reported outcomes; US: Urinary Symptoms; BS: Bowel Symptoms; TS: Hormonal-treatment-related Symptoms; SX: Sexual Functioning; CFA: Confirmatory factor analysis; GFI: Goodness-of-fit index; NFI: Bentler-Bonett normed fit index; NNFI: Non-normed fit index; CFI: Comparative fit index; RMSEA: Root mean square error of approximation; SRMR: Standard root mean-square residual; Infit: Infit mean-square; Means inlier-sensitive or information-weighted fit; DIF: Differential item functioning; PSA: Prostate-specific antigen; IIEF-15: The 15-item version of the International Index of Erectile Dysfunction; MSHQ: The Male Sexual Health Questionnaire.

## Competing interests

All authors declare that they have no competing interests.

## Authors’ contributions

CHC and WML designed the study, wrote the protocol and revised the manuscript. HCW was the coordinator of this research and conducted the field work. YJC performed the statistical analyses and drafted the manuscript. HCL, JYW and TCL designed the study, wrote the protocol and managed the field work. YCY was responsible for data collection and interpretation. All authors contributed to and have approved the final manuscript.
